# MicroRNA-18 and microRNA-19 regulate CTGF and TSP-1 expression in age-related heart failure

**DOI:** 10.1111/j.1474-9726.2011.00714.x

**Published:** 2011-10

**Authors:** Geert C van Almen, Wouter Verhesen, Rick E W van Leeuwen, Mathijs van de Vrie, Casper Eurlings, Mark W M Schellings, Melissa Swinnen, Jack P M Cleutjens, Marc A M J van Zandvoort, Stephane Heymans, Blanche Schroen

**Affiliations:** 1 Center for Heart Failure Research, Cardiovascular Research Institute Maastricht (CARIM), Maastricht UniversityMaastricht, the Netherlands; 2 Department of Pathology, Cardiovascular Research Institute Maastricht (CARIM), Maastricht UniversityMaastricht, the Netherlands; 3 Department of Biomedical Engineering, Cardiovascular Research Institute Maastricht (CARIM), Maastricht UniversityMaastricht, the Netherlands

**Keywords:** cardiac aging, connective tissue growth factor, matricellular proteins, microRNA, miR-18, miR-19, thrombospondin-1

## Abstract

To understand the process of cardiac aging, it is of crucial importance to gain insight into the age-related changes in gene expression in the senescent failing heart. Age-related cardiac remodeling is known to be accompanied by changes in extracellular matrix (ECM) gene and protein levels. Small noncoding microRNAs regulate gene expression in cardiac development and disease and have been implicated in the aging process and in the regulation of ECM proteins. However, their role in age-related cardiac remodeling and heart failure is unknown. In this study, we investigated the aging-associated microRNA cluster 17–92, which targets the ECM proteins connective tissue growth factor (CTGF) and thrombospondin-1 (TSP-1). We employed aged mice with a failure-resistant (C57Bl6) and failure-prone (C57Bl6 × 129Sv) genetic background and extrapolated our findings to human age-associated heart failure. In aging-associated heart failure, we linked an aging-induced increase in the ECM proteins CTGF and TSP-1 to a decreased expression of their targeting microRNAs 18a, 19a, and 19b, all members of the miR-17–92 cluster. Failure-resistant mice showed an opposite expression pattern for both the ECM proteins and the microRNAs. We showed that these expression changes are specific for cardiomyocytes and are absent in cardiac fibroblasts. In cardiomyocytes, modulation of miR-18/19 changes the levels of ECM proteins CTGF and TSP-1 and collagens type 1 and 3. Together, our data support a role for cardiomyocyte-derived miR-18/19 during cardiac aging, in the fine-tuning of cardiac ECM protein levels. During aging, decreased miR-18/19 and increased CTGF and TSP-1 levels identify the failure-prone heart.

## Introduction

Aging is considered a multifactorial process that is controlled by genetic and environmental factors and ultimately leads to deterioration of body and organ functions. Heart failure (HF) is a typical age-associated disease and is characterized by unique physiological and morphological changes in aged myocardium [reviewed in (Lakatta & Sollott, 2002)]. Although the rate and mechanism through which an animal or tissue ages differ among species, the constant remodeling and accumulation of the extracellular matrix (ECM) are recognized as a key feature of cardiac aging in humans, mice, and rats and contributes to the structural changes that lead to HF with advancing age (Capasso *et al.*, 1990; Barasch *et al.*, 2009; Boyle *et al.*, 2011). In particular, the expression of the ECM proteins SPARC (Bradshaw *et al.*, 2010), fibronectin (Burgess *et al.*, 2001), thrombospondin-2 (TSP-2) (Swinnen *et al.*, 2009), and connective tissue growth factor (CTGF) (Wang *et al.*, 2010) increases with aging. Both TSP-2 knockout mice and cardiomyocyte-specific CTGF transgenic animals develop spontaneous age-related cardiomyopathy (Panek *et al.*, 2009; Swinnen *et al.*, 2009), confirming a role of ECM proteins in age-related cardiac remodeling. However, the underlying mechanisms that drive the age-related gene expression of ECM molecules remain elusive.

The identification of small noncoding microRNAs (miRNAs) opened new doors for investigating the regulation of gene expression by adding another layer of control at the post-transcriptional level. MiRNAs are approximately 22 nucleotides long RNA molecules that can target mRNAs for translational repression or degradation by complementary binding to specific sequences in the protein-coding gene transcript. The first implication of miRNAs in aging was provided by a study that showed that *lin-4* and its target protein lin-14 determine longevity in *C. elegans* by affecting the insulin-like signaling pathway (Boehm & Slack, 2005). Now, miRNAs have emerged as important mediators of diverse age-associated pathologies, ranging from cancer and diabetes to neurodegenerative disorders (Grillari & Grillari-Voglauer, 2010), and increasing evidence demonstrates altered miRNA expression profiles in aging muscle, brain, lung, and liver [reviewed in (Chen *et al.*, 2010)]. In addition, miRNAs are regulated during cellular senescence, and complete loss of miRNA function caused premature senescence in embryonic fibroblasts (Mudhasani *et al.*, 2008), putting miRNAs in the forefront of cellular senescence in different organs and cell types (Chen *et al*., 2010; Grillari and Grillari-Voglauer, 2010). Their role in the heart was first suggested by expression profiling studies showing changes in the expression of specific miRNAs in failing human hearts (van Rooij *et al.*, 2006; Thum *et al.*, 2007). Further animal models proved the involvement of specific miRNAs in cardiac development, function, and under pathological conditions (van Rooij *et al.*, 2007; Zhao *et al.*, 2007; da Costa Martins *et al.*, 2010). Nevertheless, despite their proven importance in aging, cardiac disease, and development, a role for miRNAs during cardiac aging and age-related HF remains to be elucidated.

This study investigated the role of the miR-17–92 cluster in the aged heart, in view of its central role in regulation of matrix genes and in cellular aging (Dews *et al.*, 2006; Suarez *et al.*, 2008; Shan *et al.*, 2009). This cluster encodes six miRNAs (miR-17, miR-18a, miR-19a, miR-19b, miR-20a, and miR-92a-1) that are located within an 800-base pair region of human chromosome 13. Originally, the miR-17–92 cluster was linked to tumor genesis, and transcription of the cluster was found to be directly activated by the proto-oncogene c-Myc (He *et al.*, 2005) [reviewed in(van Haaften & Agami, 2010)]. The pro-oncogenic activity of miR-17–92 partially involves the regulation of the ECM proteins CTGF and thrombospondin-1 (TSP-1) by the cluster members miR-18 and miR-19, through sequence-specific targeting within the 3′-untranslated region (3′-UTR) of these gene transcripts (Supporting information Fig. S1) (Dews *et al.*, 2006). Interestingly, cardiogenesis was severely hampered in mice deficient for miR-17–92, suggesting an important role for this cluster in cardiac development (Ventura *et al.*, 2008). This, together with miR-18 and miR-19 targeting CTGF and TSP-1 and the fact that ECM proteins are crucial for healthy cardiac aging, has led us to hypothesize that these miRNAs play a role in age-related cardiac remodeling. Therefore, we investigated whether age-related changes in miR-18a, miR-19a, and miR-19b expression regulate CTGF, TSP-1, and collagen levels in rodent models of aging-associated heart failure and in the human failing heart.

## Results

### HF-prone mice develop cardiac dilation and dysfunction at old age

As a model for age-related HF in rodents, we examined age-associated changes in cardiac function in two genetically different mouse strains, i.e. C57Bl6 (HF resistant) and C57Bl6 × 129Sv (HF prone). Both strains developed normally, and echocardiographic analysis showed no differences in cardiac function at 12 and 52 weeks of age. However, fractional shortening was significantly compromised in 104-week-old HF-prone mice compared to age-matched HF-resistant hearts (Table 1). Indices of left ventricular wall thickness, LVPW and IVS, indicated progressive thinning of the LV wall with aging in HF-prone mice, followed by ventricular dilation at old age (Table 1). HF-resistant mice on the other hand had no ventricular dilation and showed a tendency to increased wall thickness with aging, resulting in a preserved cardiac function at old age (Table 1). Age-associated cardiac dysfunction in HF-prone mice was further shown by significantly higher heart, kidney, and lung weights at old age (Table 1). Thus, poor cardiac aging in HF-prone mice was characterized by a dilated cardiomyopathy-like phenotype as is seen in human HF.

**Table 1:** Echocardiographic and morphometric analysis of male mice at different ages

	HF resistant			HF prone		
		
	12 weeks *n* = 8	52 weeks *n* = 8	104 weeks *n* = 9	12 weeks *n* = 9	52 weeks *n* = 7	104 weeks *n* = 8
FS (%)	28 ± 1.5	29 ± 1.6	29 ± 1.7	28 ± 2.0	29 ± 3.5	20 ± 2.2*†‡
LVIDd (mm)	4.0 ± 0.1	4.2 ± 0.1	4.2 ± 0.1	3.8 ± 0.2	3.7 ± 0.2	4.6 ± 0.2*†
LVIDs (mm)	2.9 ± 0.1	3.0 ± 0.1	3.0 ± 0.1	2.7 ± 0.1	2.7 ± 0.2	3.7 ± 0.3*†‡
LVPWd (mm)	0.75 ± 0.03	0.84 ± 0.03*	0.90 ± 0.03*	0.99 ± 0.11	0.91 ± 0.05	0.88 ± 0.06
LVPWs (mm)	1.16 ± 0.06	1.18 ± 0.06	1.27 ± 0.04	1.40 ± 0.08‡	1.22 ± 0.04	1.02 ± 0.07*†‡
IVSd (mm)	0.72 ± 0.01	0.81 ± 0.03*	0.81 ± 0.04*	0.96 ± 0.15	0.91 ± 0.05	0.83 ± 0.03
IVSs (mm)	1.03 ± 0.01	1.22 ± 0.04*	1.18 ± 0.07	1.41 ± 0.18	1.37 ± 0.09	1.06 ± 0.06†
Heart rate (bpm)	482 ± 14	473 ± 11	493 ± 8	506 ± 15	521 ± 9‡	502 ± 25
Body weight (g)	25.0 ± 0.3	30.8 ± 0.5*	33.2 ± 0.8*†	23.5 ± 1.1	29.8 ± 0.7*	29.8 ± 0.7*‡
HW/BW ratio (mg g^−1^)	4.8 ± 0.1	4.8 ± 0.2	4.4 ± 0.1*	5.4 ± 0.2‡	5.5 ± 0.1‡	6.0 ± 0.3‡
LuW/BW ratio (mg g^−1^)	5.7 ± 0.3	5.7 ± 0.2	6.2 ± 0.2	5.9 ± 0.3	5.9 ± 0.3	7.5 ± 1.0
LiW/BW ratio (mg g^−1^)	48.0 ± 0.7	47.7 ± 1.1	48.1 ± 0.9	47.0 ± 2.8	46.6 ± 1.6	53.7 ± 3.8
KiW/BW ratio(mg g^−1^)	8.0 ± 0.2	8.7 ± 0.3	8.7 ± 0.2	8.8 ± 0.4	11.9 ± 0.3*‡	11.3 ± 0.4*‡

FS, fractional shortening; HF, Heart failure; LVIDd and LVIDs, left ventricular internal diameter in diastole and systole; LVPWd and LVPWs, left ventricular posterior wall thickness in diastole and systole; IVSd and IVSs, intraventricular septum thickness in diastole and systole; HW, heart weight; BW, body weight; LuW, lung weight; LiW, liver weight; KiW, kidney weight.

* *P*≤0.05 vs 12 weeks of age with the same genotype.

† *P*≤0.05 vs 52 weeks of age with the same genotype.

‡ *P*≤0.05 vs HF-resistant mice with the same age.

### HF-prone hearts have more interstitial fibrosis and increased levels of CTGF and TSP-1

**Fig. 1:**
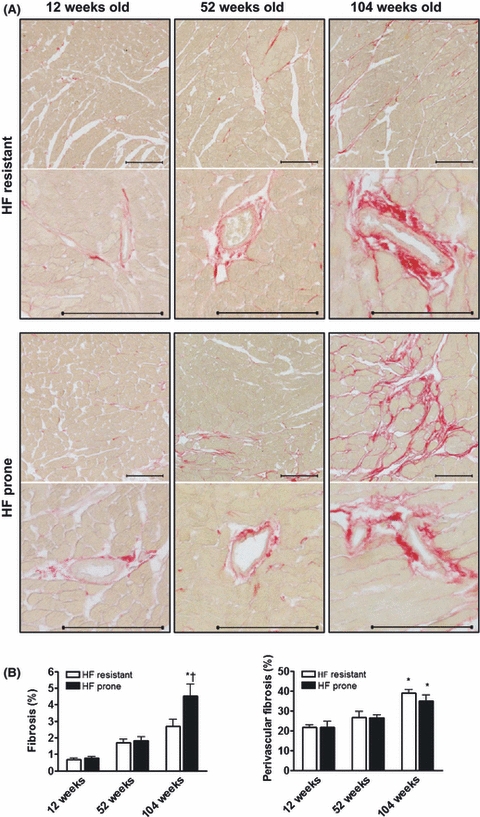
Enhanced left ventricular interstitial fibrosis in old heart failure (HF) prone mice. (A) Histological analysis of the hearts of HF-resistant and HF-prone mice by Sirius Red staining. Photographs show representative areas of interstitial fibrosis (upper panel) and collagen deposition in the perivascular area (lower panel). Scale bars represent 100 μm. (B) Quantitative analysis of the interstitial and perivascular collagen content in HF-resistant (12 weeks, *n* = 8; 52 weeks, *n* = 8; and 104 weeks, *n* = 9) and HF-prone mice (12 weeks, *n* = 6; 52 weeks, *n* = 11; and 104 weeks, *n* = 9) revealed increased interstitial fibrosis in the left ventricles of 104-week-old HF-prone mice. Perivascular fibrosis was significantly increased in 104-week-old hearts, but was not different between HF-resistant and HF-prone mice. Data are presented as mean ± SEM. *P≤0.05 vs 52-week-old HF-prone mice; †*P*≤0.05 vs 104-week-old HF-resistant mice.

Dilated cardiomyopathy is characterized by increased deposition of interstitial collagen, stiffening the heart, and compromising its contractility (Kemi *et al.*, 2000; Burgess *et al.*, 2001). Histological examination of cardiac sections showed significant interstitial fibrosis in HF-prone mice as compared to 52-week littermates and to 104-week-old HF-resistant hearts (Fig. 1A, B). On the other hand, the mild increase in collagen in aged HF-resistant mice was not significant (Fig. 1A, B), while accumulation of interstitial collagen was similar in 12- and 52-week-old HF-resistant and HF-prone mice. As expected, perivascular fibrosis increased with aging, but was not different between HF-prone and HF-resistant hearts (Fig. 1A, B).

**Fig. 2:**
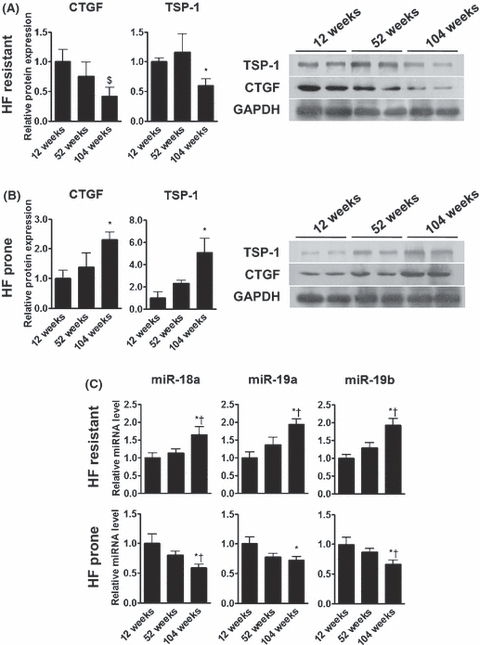
Opposite expression profiles in heart failure (HF)-resistant versus HF-prone mice. CTGF, TSP-1, miR-18a, miR-19a, and miR-19b levels in aged HF-resistant (12 weeks, *n* = 8; 52 weeks, *n* = 8; and 104 weeks, *n* = 9) and HF-prone mice (12 weeks, *n* = 6; 52 weeks, *n* = 11; and 104 weeks, *n* = 9). (A and B) Immunoblotting was performed on four mice per age-group and revealed significant induction of CTGF and TSP-1 protein expression in failing hearts of 104-week-old HF-prone mice, whereas CTGF and TSP-1 levels were reduced in old HF-resistant mice. Immunoblots show representative protein bands of TSP-1, CTGF, and GAPDH. (C) RT-PCR analysis showed increased expression of miR-18a, miR-19a, and miR-19b in 104-week-old HF-resistant hearts, whereas age-matched HF-prone mice had decreased expressions. miRNA expression and CTGF and TSP-1 protein levels were normalized for GAPDH expression and presented as mean ± SEM. *P≤0.05 vs 12 weeks of age; †*P*≤0.05 vs 52 weeks of age; $*P* = 0.05 vs 12 weeks of age.

The ECM proteins CTGF and TSP-1 are recognized as powerful regulators of ECM remodeling and are mediators of tissue fibrosis in humans, mice, and rats (Belmadani *et al.*, 2007; Shi-Wen *et al.*, 2008). We found significantly increased cardiac transcript and protein levels of CTGF and TSP-1 in 104-week-old HF-prone mice (Fig. 2A, B; Supporting information Fig. S2). *Vice versa*, the hearts of aged HF-resistant mice had no induction of CTGF and TSP-1 transcript or protein levels (Fig. 2A, B). Therefore, increased interstitial fibrosis and CTGF and TSP-1 expression characterize the HF-prone aged heart.

### Opposite cardiac miR-17–92 cluster expression profiles in HF-resistant and HF-prone aging

CTGF and TSP-1 have been identified as target genes of the miR-17–92 cluster (Dews *et al.*, 2006), more specifically of the cluster members miR-18a and miR-19a/b (Suarez *et al.*, 2008; Ohgawara *et al.*, 2009). We found opposite expression profiles of the miR-17–92 cluster in HF-prone aging compared to aging with preserved cardiac function. At 104 weeks of age, HF-prone mice had significantly reduced expression levels of miR-17, miR-18a, miR-19a, miR-19b, miR-20a, and miR-92a-1 as compared to 12-week littermates (Fig. 2C and Supporting information Table S1), coinciding with the observed increased presence of their targets TSP-1 and CTGF. Aging of HF-resistant mice, on the other hand, was accompanied by significantly enhanced expression of these miRNAs, except for miR-17 and miR-20a (Supporting information Table S1).

### The miR-18/19 – CTGF/TSP-1 axis is regulated in human age-associated heart failure

**Fig. 3:**
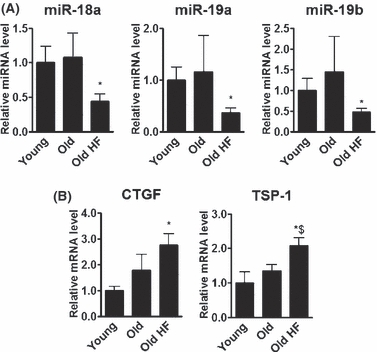
CTGF and TSP-1 expression are elevated in human heart failure (HF). RT-PCR analysis of miR-18a, miR-19a, miR-19b, CTGF, and TSP-1 transcript levels in myocardial biopsies from idiopathic cardiomyopathy (ICM) patients at older age with normal (*n* = 5) and severely impaired (*n* = 9) cardiac function. Transcript levels were compared to the expression in young ICM subjects with a preserved cardiac function (*n* = 5). (A) MiR-18a, miR-19a, and miR-19b expression was significantly decreased in older ICM patients with HF. (B) CTGF and TSP-1 transcript levels were significantly induced in older patients with a compromised cardiac function, when compared to younger ICM subjects. All data were normalized for GAPDH expression and presented as mean ± SEM. *P≤0.05 vs young ICM patients. $*P*a = 0.06 in failing vs nonfailing hearts of older ICM patients.

The three miR-17–92 cluster members miR-18a, miR-19a, and miR-19b specifically target the ECM proteins CTGF and TSP-1. To investigate the role of these genes in human HF, we studied their expression profiles in cardiac biopsies of idiopathic cardiomyopathy (ICM) patients at old age with a moderately decreased or preserved systolic function (ejection fraction (EF) between 40 and 55%) (Paulus *et al.*, 2007) and severely impaired cardiac function (EF < 30%) and compared them to young ICM subjects. In line with reduced expression levels in failing hearts of old mice, decreased miR-18a, miR-19a, and miR-19b expression was associated with severe heart failure at old age (Fig. 3A), while miRNA expression in old patients with a preserved function was not different from young ICM patients (Fig. 3A). Additionally, CTGF and TSP-1 transcript levels were significantly induced in old failing ICM hearts, which further corroborates our findings in old HF-prone mice (Fig. 3B). Together, these data suggest that regulation of CTGF and TSP-1 is the result of the shared expression of miR-18a, miR-19a, and miR19b, enabling modest changes in miRNA expression to control transcriptional repression.

### The miR-18/19 – CTGF/TSP-1 axis is regulated in aged cardiomyocytes *in vitro*

To gain further insight into the role of the miR-17–92 cluster in aging of cardiomyocytes, neonatal rat cardiomyocytes (NRCMs) were aged *in vitro*, and miRNA levels were determined.

**Fig. 4:**
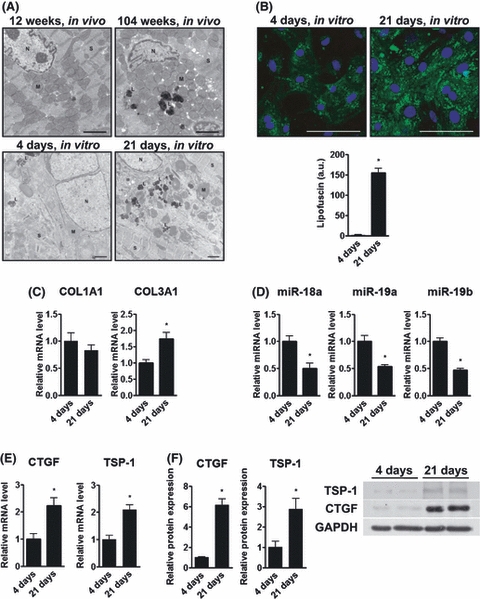
Aging-induced expression profiles in cardiomyocytes *in vitro*. (A) Electron microscopic images of the left ventricle of 12- and 104-week-old mice, and 4- and 21-day-old neonatal rat cardiomyocytes (NRCMs) showing perinuclear accumulation of lipofuscin in cardiomyocytes of 104-week-old mice and NRCMs after 21 days in culture. Scale bars represent 2 μm. (B) Two-photon/confocal images and quantification of autofluorescent lipofuscin granules (green) in cultured cardiomyocytes. Nuclei are stained with Hoechst (blue). Scale bars represent 100 μm. (C) RT-PCR analysis showed significant induction of collagen type 3A1 (COL3A1), but not collagen type 1A1 (COL1A1) in cultured NRCMs. (D) RT-PCR analysis revealed decreased miR-18a, miR-19a, and miR-19b expression in aged NRCMs after 21 days in culture. (E) CTGF and TSP-1 transcript levels increased with NRCM aging *in vitro*. (F) Immunoblotting confirmed the increase in CTGF and TSP-1 protein induction during cardiomyocyte aging. All *in vitro* experiments were performed with n = 3 per group, and protein and transcript levels were normalized for GAPDH expression. Data were presented as mean ± SEM. *P≤0.05 vs 4-day-old NRCMs.

Aging of cardiomyocytes *in vitro* was validated by comparing the lipofuscin content in 4- and 21-day-old NRCMs to the intracellular accumulation of lipofuscin in the hearts of 12- and 104-week-old C57Bl6 mice. Lipofuscin, being autofluorescent, nondegradable biological ‘garbage’, is a hallmark of aging in postmitotic cells, such as cardiomyocytes and neurons (Terman & Brunk, 2004). On high magnification examination using electron microscopy, accumulation of lipofuscin was rarely observed in the hearts of 12-week-old mice, whereas a large amount of intralysosomal lipofuscin inclusions was detected in 104-week-old mice (Fig. 4A). Prolonged culturing of NRCMs resembles the aging process in mice, as lipofuscin was hardly detected at 4 days but had accumulated at 21 days, while the characteristic sarcomere structures were maintained (Fig. 4A). Using optical imaging lipofuscin content per cardiomyocyte increased >150-fold after 21 days compared to 4-day-old NRCMs (Fig. 4B).

Beside lipofuscin accumulation, the aged myocardium is hallmarked by increased matrix deposition. To study the production of collagen in aged cardiomyocytes, we determined collagen type 1A1 and 3A1 levels. RT-PCR analysis showed significant induction of the thin collagen type 3A1, but not the thicker type 1A1, in 21-day-old NRCMs (Fig. 4C), further strengthening the aged phenotype of these cells. Taken together, prolonged culturing of NRCMs resembles two common characteristics of cardiac aging *in vivo* and was therefore used to study age-related changes in cardiomyocytes *in vitro*.

RT-PCR analysis showed that the expression levels of all members of the miR-17–92 cluster were reduced in aged cardiomyocytes, except miR-92a-1 (Fig. 4D and Supporting information Table S2). Importantly, miR-18a, miR-19a, and miR-19b were among the most strongly repressed miRNAs. Along with reduced expression of these miRNAs, CTGF and TSP-1 transcript and protein levels were significantly induced in aged cardiomyocytes (Fig. 4E, F). These findings confirm the expression profiles in aged HF-prone mice and again suggest that miR-18a, miR-19a, and miR-19b could transcriptionally repress CTGF and TSP-1 levels in cardiomyocyte aging and HF at old age.

### Cardiomyocyte CTGF and TSP-1 and collagen production are regulated by miR-18/19

**Fig. 5:**
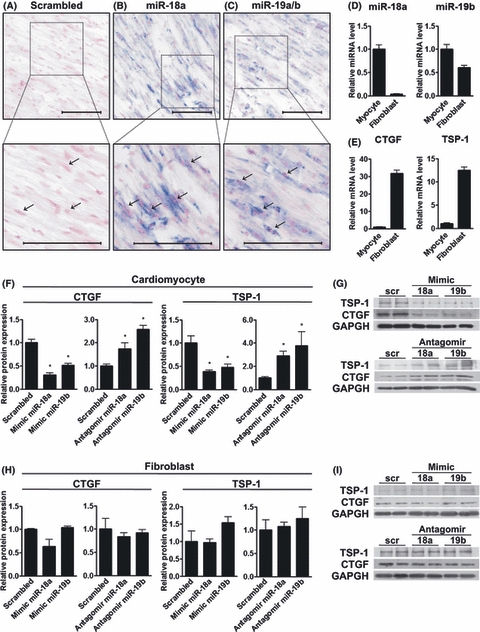
MiR-18a and miR-19b regulate CTGF and TSP-1 expression in cardiomyocytes. (A–C) *In situ* hybridization showed the abundant expression of miR-18a and miR-19b in the myocardium of adult C57Bl6 mice, most of it expressed by cardiomyocytes. Black arrows indicate the cardiomyocyte nucleus and illustrate the perinuclear localization of these miRNAs. (D and E) Comparison of the expression profiles in cultured neonatal rat cardiomyocytes (NRCMs) and neonatal rat cardiac fibroblasts (NRCFs) shows that abundant expression of miR-18a and miR-19b in NRCMs is paralleled by low CTGF and TSP-1 transcript levels. (F and G) Immunoblotting revealed that manipulating miR-18a and miR-19b function by overexpression of these miRNAs using mimics in NRCMs was sufficient to decrease CTGF and TSP-1 protein expression, while inhibition with antagomirs enhanced CTGF and TSP-1 levels. (H and I) In contrast to NRCMs, immunoblotting in cultured NRCFs showed that CTGF and TSP-1 protein expression was not suppressed by overexpression of miR-18a and miR-19b, nor did inhibition of these miRNAs result in increased CTGF and TSP-1 levels. Mimic and antagomir experiments were performed with *n* = 4 per group, and data were normalized for GAPDH expression. Data were presented as mean ± SEM. *P≤0.05 vs scrambled control oligonucleotides.

To investigate the cardiac localization of miR-18 and -19, we performed *in situ* hybridization. MiR-18a and miR-19b are abundantly expressed in the adult mouse heart and are predominantly localized in the perinuclear area of cardiomyocytes (Fig. 5A–C). This was corroborated by the finding that miR-18a and miR-19b expression was higher in cardiomyocytes compared to cardiac fibroblasts (Fig. 5D). Importantly, the abundant expression of miR-18a and miR-19b in cardiomyocytes coincides with low levels of CTGF and TSP-1, whereas in cardiac fibroblasts, relatively low levels of miR-18a and miR-19b were associated with high CTGF and TSP-1 transcription (Fig. 5E).

Next, we performed a series of functional studies to determine the role of miR-18a and miR-19b in the regulation of CTGF and TSP-1 and collagen production in cardiomyocytes and cardiac fibroblasts. Overexpression of miR-18a and miR-19b, using miRNA mimics, resulted in significant repression of CTGF and TSP-1 mRNA and protein expression in cardiomyocytes (Fig. 5F and G; Supporting information Fig. S3A). *Vice versa*, blunting of miR-18a and miR-19b using antagomirs was sufficient to increase CTGF and TSP-1 transcript and protein levels in cardiomyocytes (Fig. 5F, G; Supporting information Fig. S3A). Cardiac fibroblasts demonstrated decreased CTGF and TSP-1 transcript levels upon introduction of miR-18a and miR-19b; however, this did not result in reduced protein levels (Fig. 5H, I). Along the same line, endogenous miRNA inhibition was not sufficient to enhance CTGF and TSP-1 transcript and protein expression in cardiac fibroblasts (Fig. 5H, I; Supporting information Fig. S3B). These results show that regulation of CTGF and TSP-1 by miR-18a and miR-19b is uniquely restricted to the cardiomyocyte.

**Fig. 6:**
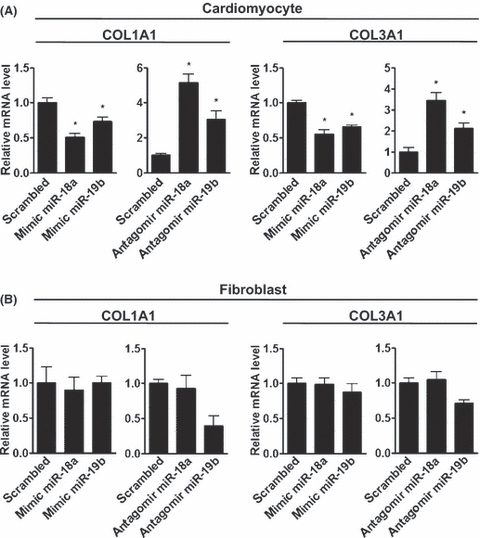
MiR-18a and miR-19b regulate collagen 1A1 and 3A1 expression in cardiomyocytes *in vitro.* RT-PCR analysis for the induction collagen 1A1 (COL1A1) and collagen 3A1 (COL3A1) in cultured neonatal rat cardiomyocytes and cardiac fibroblasts after manipulation with miR-18a and miR-19b mimics and antagomirs. (A) Overexpression of miR-18a and miR-19b in cardiomyocytes significantly reduces collagen 1A1 and collagen 3A1 transcript levels, while inhibition of these miRNAs using antagomirs significantly induced transcription of both collagen types. (B) Collagen 1A1 and 3A1 expression in cultured cardiac fibroblasts seemed unrelated to miR-18a and miR-19b, as no significant repression or induction was observed in NRCFs after treatment with miR-18a and miR-19b mimics or antagomirs, respectively. All experiments were performed with *n* = 4 per group, and data were normalized for GAPDH transcript levels. Data were presented as mean ± SEM. *P≤0.05 vs scrambled control oligonucleotides.

CTGF and TSP-1 are pro-fibrotic (Belmadani *et al.*, 2007; Shi-Wen *et al.*, 2008), and therefore, we investigated whether their regulating miRs-18a and miR-19b were also capable of affecting collagen production. Indeed, overexpression of miR18a and miR-19b in cardiomyocytes repressed collagen 1A1 and 3A1 mRNA levels, while inhibition of these miRNAs using antagomirs significantly enhanced collagen transcription (Fig. 6A). In contrast, collagen 1A1 and 3A1 transcription was not affected by either miR-18a and miR-19b overexpression or inhibition in neonatal rat cardiomyocytes and cardiac fibroblasts (NRCFs), indicating that collagen expression in cardiac fibroblasts is unrelated to these miRNAs (Fig. 6B). Thus, in concordance with CTGF and TSP-1 regulation by miR-18a and miR-19b in cardiomyocytes, these data strongly imply that miR-18a and miR-19b contribute to the induction of collagen synthesis in aged cardiomyocytes via the regulation of the pro-fibrotic CTGF and TSP-1.

## Discussion

With aging, cardiac function declines as the result of cardiomyocyte loss, left ventricular hypertrophy, dilation and accumulation and remodeling of the extracellular matrix (Lakatta & Sollott, 2002). It is well accepted that genetic changes significantly contribute to the aging process in the heart (Bodyak *et al.*, 2002; Park & Prolla, 2005). Because of the highly accessible mRNA microarray technique, most studies primarily focused on the regulation of gene expression by transcriptional control. With the discovery of miRNAs, now the emphasis is being expanded to the post-transcriptional level of regulation that gives rise to these age-specific gene profiles.

A study showing that the miRNA *lin-4* and its target protein lin-14 determine life span in *C. elegans* has paved the way for exploring the role of miRNAs in aging (Boehm & Slack, 2005). Now, increasing evidence on miRNAs as mediators of age-associated pathologies, together with reports of age-related changes in miRNA expression in aged organs and senescent cells, points toward miRNA regulation as a common biological mechanism that underlies aging and cellular senescence in different organs and cell types (Mudhasani *et al.*, 2008; Chen *et al*., 2010; Grillari and Grillari-Voglauer, 2010). Nevertheless, so far no studies have addressed the role of miRNAs in the old heart and aging of cardiomyocytes.

In the present study, we showed extensive changes in the expression of the miR-17–92 cluster in a model of age-related heart failure in mice. We were able to extrapolate our findings to patients, which confirm the well-recognized highly conserved nature of microRNAs and their target sites. Initially, the miR-17–92 cluster was linked to cancer pathogenesis and was thought to be pro-tumorgenic because of its regulation by c-Myc (Dews *et al.*, 2006). However, the cluster was later found to be tumor suppressive in multiple forms of cancer (Zhang *et al.*, 2006). Tumor suppressor mechanisms can induce cellular senescence and contribute to the aging process (Campisi, 2003), turning the miR-17–92 cluster into a potential mediator of aging. Indeed, overexpression of miR-17 and miR-20a inhibited senescence in primary human fibroblasts by blunting the activation of p21^WAF1^, while inhibition of miR-17 caused senescence in anaplastic thyroid cancer cells (Takakura *et al.*, 2008; Hong *et al.*, 2010). Furthermore, miR-17–92 expression is consistently down-regulated in multiple models of aging, i.e. after irradiation (Maes *et al.*, 2008), p53 induction (Brosh *et al.*, 2008), or stress-induced senescence (Li *et al.*, 2009), and in old human skin, bone-marrow-derived mesenchymal stem cells, T cells (Hackl *et al.*, 2010), and peripheral blood mononuclear cells (Noren Hooten *et al.*, 2010). These reports are in line with our data showing repression of the miR17–92 cluster in old failing hearts. In addition, we demonstrate that the miR-17–92 cluster is part of the senescence signature of the aged cardiomyocyte.

From the six members of the miR-17–92 cluster, miR-18a, miR-19a, and miR-19b were among the most strongly repressed miRNAs in aged cardiomyocytes and hearts of old failure-prone mice. Several studies have linked these specific cluster members, and not the other cluster members, to the matricellular proteins CTGF and TSP-1 (Dews *et al.*, 2006; Suarez *et al.*, 2008; Ohgawara *et al.*, 2009). Interestingly, CTGF upregulation in age-induced cardiac disease correlates with TGF-β induction, cardiac fibrosis, and left ventricular stiffening (Koitabashi *et al.*, 2007; Wang *et al.*, 2010). The role of TSP-1 in cardiac aging was not reported so far, but another member of the family of thrombospondins, TSP-2, was recently shown to be up-regulated in aged hearts and to protect against age-related cardiac dysfunction (Swinnen *et al.*, 2009). Here, we showed increased expression of CTGF and TSP-1 in age-related HF both in mice and human.

Traditionally, it has been assumed that CTGF and TSP-1 are predominantly expressed by fibroblasts in the heart. However, several studies have recognized that during cardiac remodeling, CTGF and TSP-1 are also secreted by cardiomyocytes to regulate their surrounding ECM environment (Ohnishi *et al.*, 1998; Chen *et al.*, 2000; Frangogiannis *et al.*, 2005). Moreover, a role for miRNAs in regulating CTGF expression in cardiomyocytes has been established by Duisters *et al.*, (2009), who showed that increased CTGF transcription during pathological LV hypertrophy in (young) hearts is controlled by miR-30 and miR-133. Our *in vitro* results support a role for miR-18a, miR-19a, and miR-19b in regulating CTGF and TSP-1 expression in the aged cardiomyocyte. Here, miRNA mimics of miR-18a and miR-19b blunted the expression of CTGF and TSP-1, and *vice versa*, inhibition of these miRNAs enhanced CTGF and TSP-1 levels. In cardiac fibroblasts, overexpression of miR-18a and miR-19b also decreased CTGF and TSP-1 transcription; however, inhibition of these miRNAs was not sufficient to increase CTGF and TSP-1. This may be attributed to the fact that a fibroblast produces large amounts of CTGF and TSP-1 while it contains relatively low amounts of miR-18a and miR-19b. These results imply that the age-related regulation of CTGF and TSP-1 expression by miR-18a and miR-19b in the heart is uniquely restricted to the cardiomyocyte to control its surrounding ECM.

In conclusion, our study is the first to show that miRNA expression of the miR-17–92 cluster changes with cardiac aging and associates decreased miR-18a, miR-19a, and miR-19b expression with age-related remodeling in the heart. Our results suggest that up-regulation of these miRNAs in the aged failure-protected heart blunts the expression of TSP-1 and CTGF to dampen the fibrotic remodeling process that contributes to the functional decline with age. Although we do not show direct regulation of CTGF and TSP-1 by these miRNAs, previous data have proven a direct mechanism (Suarez *et al.*, 2008; Ohgawara *et al.*, 2009). This study provides evidence for the involvement of miRNAs in regulating cardiac aging and identifies them as potential new therapeutic targets for the modulation of aging-induced cardiac remodeling.

## Methods

An expanded Methods section is available in the Data S1.

### Mice

Male C57Bl6 mice were obtained from Janvier (Le Genest Saint Isle, France), and mice on a C57Bl6 × 129Sv genetic background were generated within the animal facility of the University of Maastricht. All animals were housed under standard day–night rhythm and *ad libitum* conditions until 12, 52, and 104 weeks of age. Cardiac function was assessed under sedation (2% isoflurane), followed by removal of the hearts for further histological and molecular analyses. For histology, hearts were fixed in 1% paraformaldehyde, embedded in paraffin, and stained with Sirius red. For high magnification analysis, hearts were fixed in 2.5% glutaraldehyde. Electron microscopic images were made with a Philips CM100 (F.E.I., Eindhoven, The Netherlands).

This study was approved by the Institutional Animal Research Committee and conforms with the guidelines for the use of laboratory animals formulated in the Dutch law on care and use of experimental animals.

### Patients

Nineteen subjects diagnosed with ICM were included based on age and cardiac function. Five old patients (60.5 years ± 0.8) with preserved cardiac function (ejection fraction between 40 and 55%) and no signs of coronary artery disease (EF: 45.6% ± 9.2) and nine ICM patients at older age (67.0 years ± 4.3) with a severely compromised cardiac function (EF: 18.9% ± 3.0) were compared to a group of five young patients (33.2 years ± 4.4) with preserved cardiac function (EF: 46.5% ± 4.4). This study occurred in line with the recommendations of the institutional ethics committee of the University Hospital Maastricht.

### *In vitro* experiments

Neonatal rat cardiomyocytes and cardiac fibroblasts were isolated from 1- to 3-day-old Lewis rats as described previously (De Windt *et al.*, 1997).

For aging of cardiomyocytes *in vitro,* NRCMs were cultured for 4 and 21 days, and autofluorescent lipofuscin was excited using a laser at 488 nm, with confocal detection at 510–560 nm (Nikon Eclipse E600FN, Tokyo, Japan). Hoechst-stained nuclei were excited with a two-photon Spectra-Physics Tsunami laser (Spectra-Physics, Irvine, CA, USA) centered at 800 nm and visualized at 400–480 nm. The lipofuscin content per cardiomyocyte was determined using Leica Qwin image processing and analysis software (Leica. Microsystems Cambridge Ltd, Cambridge, UK).

For the overexpression or inhibition of miR-18a and miR-19b, NRCMs and NRCFs were transfected with 80 nm miRIDIAN hairpin inhibitor (antagomiR) miR-18a (#IH-300487-06) or miR-19b (#IH-300489-05), or with miRIDIAN mimic miR-18a (#C-300487-05), or miR-19b (#C-300489-03) (Dharmacon, Colorado, CO, USA). Cells transfected with miRIDIAN microRNA hairpin inhibitor negative control #1 (#IN-001005-01) and miRIDIAN microRNA mimic negative control #2 (#CN-002000-01) served as control groups.

### RNA isolation and Real-time PCR

Total RNA was extracted from homogenized cells and heart tissues using the mirVANA miRNA isolation kit (Ambion, Austin, TX, USA) according to the manufacturer's protocol. Gene transcript levels of CTGF, TSP-1, collagen 1A1, and collagen 3A1 (primer sequences are listed in Supporting information Table S3), or miRNA expression were detected with the MyIQ Single Color Real-Time PCR detection System (Bio-Rad, Hercules, CA, USA). MiR-17, miR18a, miR-19a, miR-19b, miR-20, and miR-92a-1 expression was measured using miRNA-specific miScript Primer Assays (Qiagen, Hilden, Germany). All expression levels were presented relative to glyceraldehydes-3-phosphate dehydrogenase (GAPDH).

### Immunoblotting

Proteins were extracted from left ventricular heart tissue and *in vitro* from NRCM and NRCFs. Protein lysates were separated by SDS–PAGE and transferred to a polyvinylidene fluoride membrane (Immobilon-P, 0.45 μm pore size). The membranes were probed overnight at 4°C with a primary antibody to detect CTGF (#GTX26992; GeneTex Inc., Irvine, CA, USA), TSP-1 (in-house rabbit anti-human TSP-1 was kindly provided by MF Hoylaerts (Moura *et al.*, 2008), University of Leuven, Belgium), and glyceraldehyde-3-phosphate dehydrogenase (GAPDH) (#RDI-TRK5G4-6C5; Fitzgerald Inc., Concord, MA, USA). Protein levels were determined using Quantity One software (Bio-Rad, Hercules, CA, USA) and presented relative to GAPDH protein expression.

### *In Situ* Hybridization

Mouse left ventricular heart tissue was used for miRNA *in situ* hybridization as described previously (Nuovo, 2010). Double DIG-labeled locked nucleic acid (LNA) hybridization probes complementary to mouse mature miR-18a (5DIGN/CTATCTGCACTAGATGCACCTTA/3DIG_N) (#38462-15), miR-19b (5DIGN/TCAGTTTTGCATGGATTTGCACA/3DIG_N) (#38092-15), and a scrambled probe (5DIGN/GTGTAACACGTCTATACGCCCA/3DIG_N) (#99004-15) were purchased from Exiqon (Vedbaek, Denmark).

### Statistical analysis

All data are expressed as mean ± standard error of the mean (SEM). Differences between groups were evaluated by Student's *t*-test or one-way anova with Bonferroni post hoc test when appropriate. Differences in interstitial and perivascular fibrosis were analyzed by two-way anova and Bonferroni post hoc test. Probability values <0.05 were considered statistically significant.

## Supporting Information

**Fig. S1** CTGF and TSP-1 3′-UTRs have conserved miR-18 and miR-19 target sites.

**Fig. S2** CTGF and TSP-1 transcript levels in HF resistant and HF prone mice.

**Fig. S3** CTGF and TSP-1 transcripts are regulated by miR-18a and miR-19b in cardiomyocytes.

**Table S1** Fold change expression profiles of miR-17–92 cluster members in HF resistant and HF prone mice at different ages.

**Table S2** Fold change expression profiles of miR-17–92 cluster members in cardiomyocytes *in vitro*.

**Table S3** Real-Time PCR primers.

**Data S1** Methods.

As a service to our authors and readers, this journal provides supporting information supplied by the authors. Such materials are peer-reviewed and may be re-organized for online delivery, but are not copy-edited or typeset. Technical support issues arising from supporting information (other than missing files) should be addressed to the authors.
