# Supplementary material for: MicroRNA-18 and microRNA-19 regulate CTGF and TSP-1 expression in age-related heart failure
Source: Aging Cell. 2011 Oct;10(5):769–79. doi: 10.1111/j.1474-9726.2011.00714.x (PMC3193380; doi:10.1111/j.1474-9726.2011.00714.x)
Supplement: Supplementary file 4 [file acel0010-0769-SD4.doc]

**SUPPLEMENTAL MATERIAL**

**MicroRNA-18 and -19 regulate CTGF and TSP-1 expression in age-related heart failure**

by

Geert C. van Almen et al.

**Detailed Methods**

**Mice**

Male C57Bl6 mice were obtained from Janvier (Le Genest Saint Isle, France) and mice on a mixed genetic background (C57Bl6 × 129Sv) were backcrossed four times into the C57Bl6 background within the animal facility of the University of Maastricht. All animals were housed under standard day-night rhythm and ad libitum conditions until 12, 52 and 104 weeks of age. Cardiac function was assessed at the end of the experiment under sedation (2% isoflurane) using a 12-MHz transducer (Hewlett Packard) on a Sonos 5500 (Hewlett Packard) echocardiograph. Echocardiographic images were taken in B-mode to analyse fractional shortening (FS), left ventricular diameters at end-diastole (LVEDd) and end-systole (LVEDs), left ventricular posterior wall at end-diastole (LVPWd) and end-systole (LVPWs), and intraventricular septal thickness at end-diastole (IVSd) and end-systole (IVSs). Subsequent to echocardiography, hearts were taken out and prepared for molecular, histological and ultrastructural analysis.

The present study was approved by the Institutional Animal Research Committee and conforms with the guidelines for the use of laboratory animals formulated in the Dutch law on care and use of experimental animals.

**Histology and electron microscopy**

After removal, hearts were cut transversely, fixed in 1% buffered paraformaldehyde and embedded in paraffin for histological examination. To measure collagen in the hearts of 12, 52, and 104 weeks old mice, four µm thick paraffin sections were stained with Sirius red. Interstitial collagen content was determined as percentage Sirius red staining per total cardiac tissue area, and perivascular fibrosis was presented as percentage collagen in the area adjacent to the vasculature. All morphometric analyses were performed using a Leica DMRXE microscope (Leica Microsystems Cambridge Ltd) in combination with Leica Qwin image processing and analysis software (Leica. Microsystems Cambridge Ltd).

For high magnification images, hearts were fixed in 2.5% glutaraldehyde in 0.1 M phosphate buffer at pH 7.4, and post-fixed in 1% osmiumtetroxide in 0.067 M cacodylate buffer. After fixation, the samples were routinely dehydrated in graded ethanol series (70%, 90% and 100%), followed by embedding in Epon. Electron microscopic images were made from 80 nm thick sections with a Philips CM100 (F.E.I., Eindhoven, The Netherlands) at an accelerating voltage of 80KV.

**Patients**

Nineteen subjects diagnosed with idiopathic cardiomyopathy (ICM) were included based on age and cardiac function. Five old patients (60.5 years ± 0.8) with preserved cardiac function (ejection fraction between 40 and 55 percent) and no signs of cornary artery disease (ejection fraction (EF): 45.6 % ± 9.2), and nine ICM patients at older age (67.0 years ± 4.3) with a severely compromised cardiac function (EF: 18.9 % ± 3.0) were compared to a group of five young patients (33.2 years ± 4.4) with preserved cardiac function (EF: 46.5 % ± 4.4). All patients had no unstable angina, and no history of myocardial infarction or left ventricular hypertrophy. Three transmural needle biopsies were taken from the left ventricular anterior wall, and snap-frozen in liquid nitrogen for further molecular analysis. This study occurred in line with the recommendations of the institutional ethics committee of the University Hospital Maastricht.

**Neonatal cardiac cell isolation**

Neonatal rat cardiomyocytes (NRCMs) and cardiac fibroblasts (NRCFs) were isolated from 1- to 3-day-old Lewis rats as described previously (De Win*dt et a*l., 1997). Briefly, cardiac cells were isolated by collagenase digestion from the ventricles. After 60 minutes of preplating, the non-attached NRCMs were separated from the NRCFs and plated in gelatinized six-well plates and cultured overnight in Dulbecco’s modified Eagle’s medium (DMEM) (Invitrogen, Carlsbad, CA, USA) supplemented with 10% horse serum (PAA, Pasching, Austria), 5% heat-inactivated newborn calf serum (PAA, Pasching, Austria), M-199 (Invitrogen, Carlsbad, CA, USA), glucose (Merck, Darmstadt, Germany), gentamycin (Invitrogen, Carlsbad, CA, USA), and Penicillin Streptomycin (Invitrogen, Carlsbad, CA, USA). NRCMs were cultured in the presence of cytosine β-D-arabinofuranoside (Sigma, St Louis, MO, USA) to suppress proliferation of non-cardiomyocytes. NRCFs were cultured in DMEM (Invitrogen, Carlsbad, CA, USA), supplemented with 10% fetal bovine serum (PAA, Pasching, Austria) and gentamycin (Invitrogen, Carlsbad, CA, USA).

**Lipofuscin accumulation in aged cardiomyocytes *in vitro.***

After isolation, 7.5×10^5^ NRCMs per well were plated in six well plates and cultured for 4 and 21 days. Cardiomyocytes were cultured under high serum conditions (10% horse serum and 5% heat-inactivated newborn calf serum) as described in the section above. The medium was changed 24 hours after isolation and then every two days.

After 4 and 21 days, lipofuscin accumulation was determined using a combination of confocal and two-photon microscopy. Briefly, the medium was removed and NRCMs were incubated for 5 minutes with prewarmed, 37 degrees phosphate buffered saline (PBS) supplemented with 1 µM Hoechst 33342 (Fluka, Sigma–Aldrich Corp., St. Louis, MO, USA). Images were captured with an Eclipse E600FN upright microscope (Nikon, Tokyo, Japan) equipped with a Radiance 2100MP optical imaging system (Bio-Rad, Hercules, CA, USA). Hoechst-stained nuclei were excited with a two-photon Spectra-Physics Tsunami laser (Spectra-Physics, Irvine, CA, USA) centered at 800 nm and visualized at 400-480 nm. Subsequently, autofluorescent lipofuscin was excited using a laser at 488 nm, with confocal detection at 510-560 nm. Images were created with LaserSharp 5.0 software (Bio-Rad, Hercules, CA, USA) and lipofuscin accumulation per cardiomyocyte was determined using Leica Qwin image processing and analysis software (Leica. Microsystems Cambridge Ltd, Cambridge, UK). After measuring the lipofuscin content cells were pelleted and snap frozen in liquid nitrogen for RNA extraction.

**Transfection of cultured cardiomyocytes and cardiac fibroblasts**

For miRNA mimic (overexpression) or antagomir (inhibition) transfection, 7.5×10^5^ NRCMs and 1.5×10^5^ NRCFs per well were plated in a six-well plate and incubated overnight. The next day cells were placed on DMEM under low serum conditions for 24 hours before the medium was changed to serum-free DMEM without antibiotics for 4 hours. NRCMs and NRCFs were then transfected with 80 nm miRIDIAN hairpin inhibitor miR-18a (#IH-300487-06), miRIDIAN mimic miR-18a (#C-300487-05), miRIDIAN hairpin inhibitor miR-19b (#IH-300489-05), miRIDIAN mimic miR-19b (#C-300489-03), miRIDIAN microRNA hairpin inhibitor negative control #1 (#IN-001005-01), and miRIDIAN microRNA mimic negative control #2 (#CN-002000-01) (Dharmacon, Colorado, CO, USA), fascilitated by lipofectamine 2000 (Invitrogen, Carlsbad, CA, USA) according to the manufacturer’s protocol. After 48 hours, cells were trypsinized, pelleted, and snap frozen in liquid nitrogen for RNA isolation.

**RNA isolation and Real-Time PCR**

Total RNA was extracted from homogenized cells and heart tissues using the mirVANA miRNA isolation kit (Ambion, Austin, TX, USA) according to the manufacturer’s protocol followed by removal of genomic DNA with the DNA-free ambion kit (Ambion, Austin, TX, USA). cDNA was generated from 500 nanograms of total RNAwith the qScript cDNA synthesis kit (Quanta BioSciences Inc., Gaithersburg, MD, USA) and miScript reverse transcription kit (Qiagen, Hilden, Germany) for the detection of gene transcript levels or miRNA expression respectively. Five nanograms of cDNA template was amplified with the MyIQ Single Color Real-Time PCR detection System (Bio-Rad, Hercules, CA, USA) to measure transcript levels of CTGF, TSP-1, collagen 1A1 and collagen 3A1 (primer sequences are listed in supplemental table 2), and miRNA expression of miR-17, miR18a, miR-19a, miR-19b-1, miR-20, and miR-92a-1 using the specific miScript Primer Assays (Qiagen, Hilden, Germany). Expression levels were analyzed with BioRad iQ5 software (Bio-Rad, Hercules, CA, USA) and presented relative to glyceraldehydes-3-phosphate dehydrogenase (GAPDH).

**Immunoblotting**

Left ventricular heart tissue was homogenized (RyboLyser, Hybaid, UK) and extracted in homogenization buffer (50 mM Tris-HCl pH 7.5, 10 mM CaCl_2_, and 10 µM ZnCl_2_), diluted in 2x sample buffer (0.5 M Tris-HCl pH6.8, 20% glycerol, 4% SDS, 1 mg bromophenol blue, H_2_O, and 2-mercaptoethanol) and boiled for 5 minutes at 95˚C. NRCMs and NRCFs were harvested in a identical manner. The protein lysates (10 µg of protein) were separated by SDS-PAGE (10% running, 4% stacking) and transferred to a polyvinylidene fluoride membrane (Immobilon-P, 0.45 µm pore size). After blocking in 3.5% protifar plus (Nutricia), the membranes were probed overnight at 4˚C with a primary antibody to detect CTGF (dilution 1:500) (GeneTex Inc., Irvine, CA, USA; #GTX26992), TSP-1 (5 µg/ml) (in-house rabbit anti-human TSP-1 was kindly provided by MF Hoylaerts (Mou*ra et a*l., 2008), University of Leuven, Belgium), and glyceraldehyde-3-phosphate dehydrogenase (GAPDH) (dilution 1:10000) (Fitzgerald Inc., Concord, MA, USA; #RDI-TRK5G4-6C5). Pending on the origin of the first antibody, membranes were incubated with a horseradish-linked polyclonal goat anti-rabbit (dilution 1:2000) (Cell Signaling Technology, Danvers, MA, USA; #7074) or rabbit anti-mouse antibody (dilution 1:2000) (DAKO, Glostrup, Denmark; #P0161) followed by visualization of the signal using Enhanced Chemi Luminiscence. Protein levels were determined using Quantity One software (Bio-Rad Laboratories Inc.) and presented relative to GAPDH protein expression.

**In Situ Hypbridization**

Mouse left ventricular heart tissue was fixed in 4 percent paraformaldehyde for eight hours and embedded in tissuetek. Next, twelve µm thick cryosections were cut, dried to the air, and pretreated with pepsin (1.3 mg/ml) for twenty minutes prior to miRNA in situ hybridization as described previously (Nuovo, 2010). Double DIG-labeled Locked Nucleic Acid (LNA) hybridization probes complementary to mouse mature miR-18a (5DIGN/CTATCTGCACTAGATGCACCTTA/3DIG_N) (#38462-15), miR-19b (5DIGN/TCAGTTTTGCATGGATTTGCACA/3DIG_N) (#38092-15), and a scrambled probe (5DIGN/GTGTAACACGTCTATACGCCCA/3DIG_N) (#99004-15) were purchased from Exiqon (Vedbaek, Denmark). Probes were used at a concentration of 200 mM/µl.

**Statistical analysis**

All data are expressed as mean ± standard error of the mean (SEM). Differences between groups were evaluated by Student’s *t*-test or one-way ANOVA with Bonferroni post-hoc test when appropriate. Differences in interstitial and perivascular fibrosis were analyzed by two-way ANOVA and Bonferroni post-hoc test. Probability values <0.05 were considered statistically significant.

**Supplemental Tables**

**Supplemental Table 1**. Fold change expression profiles of miR-17~92 cluster members in HF resistant and HF prone mice at different ages

|  | **HF resistant** | | | **HF prone** | | |
| --- | --- | --- | --- | --- | --- | --- |
|  | **12 weeks**  **n=8** | **52 weeks**  **n=8** | **104 weeks**  **n=9** | **12 weeks**  **n=6** | **52 weeks**  **n=11** | **104 weeks**  **n=8** |
| **miR-17-92 cluster** |  |  |  |  |  |  |
| **miR-17** | 1.00 ± 0.13 | 0.78 ± 0.06 | 0.83 ± 0.05 | 1.00 ± 0.10 | 0.81 ± 0.05 | 0.62 ± 0.07*†‡ |
| **miR-18a** | 1.00 ± 0.14 | 1.14 ± 0.11 | 1.65 ± 0.22* | 1.00 ± 0.16 | 0.80 ± 0.07‡ | 0.59 ± 0.06*†‡ |
| **miR-19a** | 1.00 ± 0.16 | 1.36 ± 0.21 | 1.93 ± 0.15*† | 1.00 ± 0.12 | 0.78 ± 0.06‡ | 0.72 ± 0.06*‡ |
| **miR-19b-1** | 1.00 ± 0.10 | 1.28 ± 0.15 | 1.92 ± 0.19*† | 1.00 ± 0.12 | 0.87 ± 0.06‡ | 0.66 ± 0.07*†‡ |
| **miR-20a** | 1.00 ± 0.12 | 0.81 ± 0.06 | 0.86 ± 0.03 | 1.00 ± 0.11 | 0.89 ± 0.07 | 0.72 ± 0.07* |
| **miR-92a-1** | 1.00 ± 0.09 | 0.88 ± 0.07 | 1.31 ± 0.06*† | 1.00 ± 0.08 | 0.75 ± 0.05* | 0.68 ± 0.05*‡ |

**P*≤0.05 vs 12 weeks of age with the same genotype; †*P*≤0.05 vs 52 weeks of age with the same genotype; ‡ *P*≤0.05 vs HF resistant mice with the same age

**Supplemental Table 2. Fold change expression profiles of miR-17~92 cluster members in cardiomyocytes *in vitro***

|  | **4 days** | **21 days** |
| --- | --- | --- |
| **miR-17-92 cluster** |  |  |
| **miR-17** | 1.00 ± 0.19 | 0.78 ± 0.01 |
| **miR-18a** | 1.00 ± 0.10 | 0.50 ± 0.10* |
| **miR-19a** | 1.00 ± 0.11 | 0.54 ± 0.03* |
| **miR-19b-1** | 1.00 ± 0.06 | 0.47 ± 0.03* |
| **miR-20a** | 1.00 ± 0.03 | 0.77 ± 0.002* |
| **miR-92a-1** | 1.00 ± 0.08 | 1.04 ± 0.05 |

**P*≤0.05 vs 4 days old cardiomyocytes

**Supplemental Table 3. Real-Time PCR primers**

|  |  | **Primer sequence 5’-3’** | **Species** |
| --- | --- | --- | --- |
| **Gene** |  |  |  |
| **CTGF** | F | cacagagtggagcgcctgttc | Human, mouse, rat |
|  | R | gatgcactttttgcccttcttaatg |  |
| **TSP-1** | F | ggcaaggactgcgttggt | Human |
|  | R | cacttcacgccggcaaag |  |
|  | F | gactcgggacccatctatga | Mouse |
|  | R | ggttatgattggcagctgatg |  |
|  | F | gtgacggaaaatcaagtttgca | Rat |
|  | R | acttggcaccagcaaagca |  |
| **COL1A1** | F | ccgcccgcacatgc | Rat |
|  | R | ctccatgttgcagtagaccttgat |  |
| **COL3A1** | F | ggcaatgtaaagaagtctctgaagct | Rat |
|  | R | gtgtttgatattcaaagactgtcttgct |  |
| **GAPDH** | F | ccacccatggcaaattcc | Human |
|  | R | gggatttccattgatgacaag |  |
|  | F | ggtggacctcatggcctaca | Mouse |
|  | R | ctctcttgctcagtgtccttgct |  |
|  | F | ggtggacctcatggcctaca | Rat |
|  | R | ctctcttgctctcagtatccttgct |  |

CTGF, connective tissue growth fator; TSP-1, thrombospondin-1; COL1A1, collagen 1A1; COL3A1, collagen 3A1; GAPDH, glyceraldehyde-3-phosphate dehydrogenase. F indicates forward primer and R indicates reverse primer.

**Supplemental References**

De Windt LJ, Willemsen PH, Popping S, Van der Vusse GJ, Reneman RS, Van Bilsen M (1997) Cloning and cellular distribution of a group II phospholipase A2 expressed in the heart. *J Mol Cell Cardiol*. **29**, 2095-2106.

Moura R, Tjwa M, Vandervoort P, Van Kerckhoven S, Holvoet P, Hoylaerts MF (2008) Thrombospondin-1 deficiency accelerates atherosclerotic plaque maturation in ApoE-/- mice. *Circ Res*. **103**, 1181-1189.

Nuovo GJ (2010) In situ detection of microRNAs in paraffin embedded, formalin fixed tissues and the co-localization of their putative targets. *Methods*. **52**, 307-315.

**Supplemental figure legends**

**Supplemental figure 1. CTGF and TSP-1 3’-UTRs have conserved miR-18 and miR-19 target sites.** (A) Diagram indicating the location of the target sequences of miR-18 and miR-19 in the human CTGF and TSP-1 gene. (B) MiRNA-target hybrids of the miR-17~92 cluster members miR-18a, miR-19a, and miR-19b with their target sequences, based on prediction by TargetScan Human Release 5.1 ([www.targetscan.org](http://www.targetscan.org/)). (C) Alignment of the target sites for miR-18 and 19 found in the 3’-UTR region of the CTGF and TSP-1 gene of human, mouse and rat, illustrating that these sites are highly conserved among species. The 3’UTR region of the TSP-1 gene is not completely annotated in rat, and therefore the miR-19 target site in the rat TSP-1 gene is currently unknown. (D) Sequence alignment of the mature forms of miR-18a, miR-19a, and miR-19b shows that each of these miRNAs are identical in human, mouse and rat. MiR-19a and –b only differ in one nucleotide outside the seed sequence.

**Supplemental figure 2. CTGF and TSP-1 transcript levels in HF resistant and HF prone mice.** CTGF and TSP-1 transcript levels were determined in aged HF resistant (12 weeks, n=8; 52 weeks, n=8; and 104 weeks, n=9) and HF prone mice (12 weeks, n=6; 52 weeks, n=11; and 104 weeks, n=9).

RT-PCR analysis showed enhanced CTGF and TSP-1 mRNA levels in the hearts of 104 weeks old HF prone mice, but not in HF resistant hearts. All data were normalized for GAPDH expression and presented as mean ± SEM. **P*≤0.05 vs 12 weeks of age; †*P*≤0.05 vs 52 weeks of age.

**Supplemental figure 3. CTGF and TSP-1 transcripts are regulated by miR-18a and miR-19b in cardiomyocytes.** Transcript levels of CTGF and TSP-1 were determined in NRCMs and NRCFs after transfection with a miRNA-specific mimic or antagomir to overexpress or inhibit, respectively, the function of miR-18a and miR-19b. (A) RT-PCR analysis revealed that overexpression of miR-18a and miR-19b in cardiomyocytes significantly repressed CTGF and TSP-1 transcription, while inhibition of these miRNAs enhanced CTGF and TSP-1 mRNA levels. (B) Although overexpression of miR-18a and miR-19b decreased CTGF and TSP-1 expression, blunting of the miRNAs using antagomirs was not sufficient to increase CTGF and TSP-1 levels in cardiac fibroblasts. Mimic and antagomir experiments were performed with n=4 per group and data were normalized for GAPDH expression. Data were presented as mean ± SEM.
